# Multimodal Deep Learning Model for Cylindrical Grasp Prediction Using Surface Electromyography and Contextual Data During Reaching

**DOI:** 10.3390/biomimetics10030145

**Published:** 2025-02-27

**Authors:** Raquel Lázaro, Margarita Vergara, Antonio Morales, Ramón A. Mollineda

**Affiliations:** 1 Department of Mechanical Engineering and Construction, Universitat Jaume I, 12071 Castelló, Spain; raquel.lazaro@uji.es; 2 Department of Computer Science and Engineering, Universitat Jaume I, 12071 Castelló, Spain; morales@uji.es; 3 Institute of New Imaging Technologies, Universitat Jaume I, 12071 Castelló, Spain; mollined@uji.es

**Keywords:** EMG, hand grasp prediction, machine learning, multimodal data fusion

## Abstract

Grasping objects, from simple tasks to complex fine motor skills, is a key component of our daily activities. Our approach to facilitate the development of advanced prosthetics, robotic hands and human–machine interaction systems consists of collecting and combining surface electromyography (EMG) signals and contextual data of individuals performing manipulation tasks. In this context, the identification of patterns and prediction of hand grasp types is crucial, with cylindrical grasp being one of the most common and functional. Traditional approaches to grasp prediction often rely on unimodal data sources, limiting their ability to capture the complexity of real-world scenarios. In this work, grasp prediction models that integrate both EMG signals and contextual (task- and product-related) information have been explored to improve the prediction of cylindrical grasps during reaching movements. Three model architectures are presented: an EMG processing model based on convolutions that analyzes forearm surface EMG data, a fully connected model for processing contextual information, and a hybrid architecture combining both inputs resulting in a multimodal model. The results show that context has great predictive power. Variables such as object size and weight (product-related) were found to have a greater impact on model performance than task height (task-related). Combining EMG and product context yielded better results than using each data mode separately, confirming the importance of product context in improving EMG-based models of grasping.

## 1. Introduction

Using one’s hands is essential for daily activities, from holding a bottle to complex tasks like threading a needle. Though these are quite different actions, both begin with the reach-to-grasp movements involving the hand adjusting to an object’s size and shape [1]. The selection of grasp types is influenced by a combination of cognitive and biomechanical factors, depending on the task, object properties, and environmental constraints [2]. Studies have shown that object size, task constraints, and object mass are the best predictors of grasp type, with 47% accuracy [3]. Grasp analysis has been extensively studied with the goal of accurately analyzing or generating natural movements to improve human–machine interaction [4]. These efforts focus on enhancing the functionality of artificial hands or arms and developing autonomous systems that mimic human grasping.

The use of EMG for pattern recognition in robotics [5], prosthetics [6], and exoskeletons is popular because surface EMG is easy to collect and does not affect the user’s performance and is not compromised by other data sources, namely kinematics or video-based methods [7]. According to [8], 22% of hand exoskeletons (HEs) are controlled using explicit or implicit signals like EMG.

The main models used to classify movements of hands and fingers from EMG sources [9] are Support Vector Machines (39%), Convolutional Neural Networks (CNNs) (24%), Artificial Neural Networks (24%), K-Nearest Neighbors (21%), Linear Discriminant Analysis (15%), and Deep Neural Networks (12%). Common machine learning metrics, like accuracy, precision, and specificity, are used to assess model performance. In EMG-based classification, key challenges include noise in muscle signals, electrode placement, and electrode type [10]. Electrode displacement between sessions, even with the same subject, impacts data reliability and model consistency [11]. Some approaches use cross-validation to prevent these errors [12]. In [13], grasping and releasing are detected for several surface EMG placements to create a robust model that predicts grasp intention.

Some attempts have been made to detect the user’s intent to grasp earlier in the motion, to enable faster, more seamless prosthetic control aligned with the user’s natural movements. Typically, EMG is extracted from the forearm, but there are solutions that also include the muscles of the whole arm [14]. In [15], the reach-to-grasp movement is divided into three phases based on the relative movement of the forearm to the upper arm. Several studies present combinations of EMG with other types of data. In [16], EMG and kinematic data from vision modules were used to predict finger joint angles and grasp types. This methodology, along with attention mechanisms that help us to focus only on the important sections of the data, is highlighted for online pattern recognition. For fast pattern recognition, a short initial segment of EMG signals (100ms) has also proven to be effective to identify pinching or grasping [17]. Another technique that combines EMG and images of the objects to be grasped also showed promising results for five grasp types when applying transfer learning to existing models [18]. The synergies between several data sources have proven to be effective in terms of enhancing these types of models.

Furthermore, EMG data processing models are typically very complex, as they usually involve intricate architecture or segmentation and feature extraction processes (FEs) [19], in addition to signal processing techniques. Although FEs are a valid form of processing the data, one of the key benefits of using neural networks is their ability to learn features automatically from raw data. In the case of EMG, if the noise has been filtered out, the networks could learn inherent patterns of the signal that would likely have been missed in a manual FE process. In this field, a study found a simple architecture based on one-dimensional convolutions to process EMG signals during grasps without FE [20].

In addition, many of the existing solutions use data from hand postures instead of real grasping actions. Databases such as NinaPro [21,22] collect information on several grasping actions and have been used to detect grasp types during activities similar to those in real environments with models such as a multi-layer perceptron [23]. Unfortunately, these datasets only represent grasping motions that are not followed by any other action. For reliable and robust models, it is interesting to use data extracted from activities of daily living (ADL), based on reach-to-grasp movements followed by other actions such as object transportation or interaction with the environment.

The aim of our work is to incorporate ADL into a model with a simple architecture to perform pattern recognition based on surface EMG data without explicit FEs, and assess whether incorporating contextual data pertaining to the height of the task or weight of the product can enhance the results. For this purpose, unimodal (EMG-based or context-based) and multimodal approaches are compared. Furthermore, the contribution of each contextual variable included in the multimodal model is evaluated and discussed.

## 2. Materials and Methods

### 2.1. Dataset

**Figure 1:**
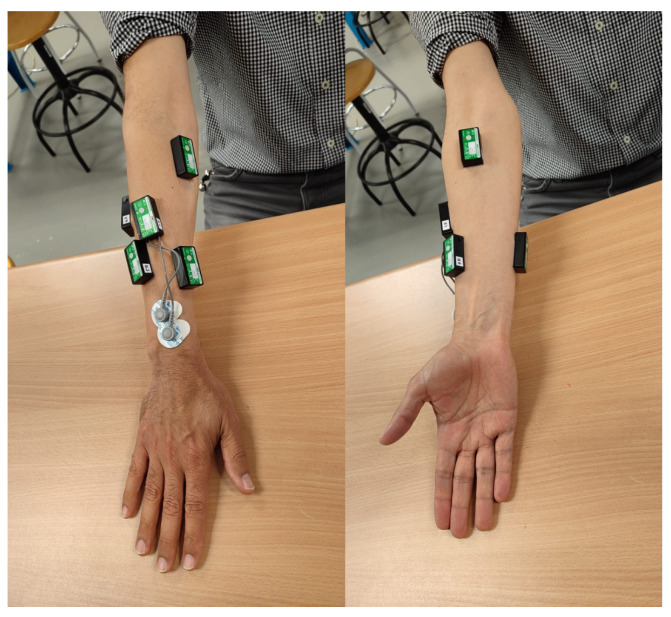
Seven zones for surface EMG placement from [24].

The data used for this work can be found in MOVMUS-UJI dataset [24]. This dataset contains kinematic data of the hand, surface EMG of the forearm muscles, as well as contextual information about the product weight, task height, gender of the participant, etc., in more than 150 tasks of ADL. In this work, only EMG signals, task and product information, and annotations regarding grasp type have been used. The EMG data were recorded from seven distinct zones on the forearm (Figure 1), which were chosen as the most representative of forearm muscles based on prior research [25] and in accordance with SENIAM guidelines [26]. The equipment used included a Biometrics DataLITE wireless recording unit (Biometrics Ltd., Newport, UK) and seven wireless electrodes (LE230).

Signal filtering involved applying a fourth-order band-pass filter between 25–500 Hz, rectification, and a subsequent fourth-order low-pass filter at 8 Hz, followed by Gaussian smoothing for further refinement, as outlined in [27]. To process the signals, the data were normalized by subject, using maximum voluntary contraction (MVC) values obtained from seven different movements: finger flexion and extension, wrist flexion and extension, ulnar and radial deviation of the wrist, and forearm pronation. Each EMG recording was divided by the maximum value across these MVCs or any recorded activity performed by the participant, so that values are comparable across subjects and sessions.

In the dataset, a task corresponds to a single recording of a predefined sequence of actions, during which a human operator marked events to divide the recording into distinct elementary tasks (ETs) such as holding, pouring, reaching, twisting, and so on. These event markers allowed the whole recording to be segmented into different actions. Table 1 illustrates two examples of how a task was divided into elementary tasks. For the purpose of this work, each action or ET will be called a recording. Data were collected in three phases: Phase A involved 79 tasks and 342 ETs with right-handed grasp types, including cylindrical, lateral pinch, and lumbrical grasps; Phase B involved 33 tasks and 128 ETs with right-handed grasp types such as oblique, special pinch, hook, intermediate, and five-finger pinch; Phase C involved 49 bimanual tasks and 144 ETs, encompassing all grasp types.

Thirty right-handed participants, evenly distributed by gender and aged between 20 and 55 years, and free of upper limb pathology were recruited for the study. The mean age of the participants was 30.8 ± 11.2 years in Phase A and 31.2 ± 11.2 years in Phases B and C.

Table 2 contains the variables that have been used in this work and the modifications performed on some of the variables that are explained in the data preparation section. For each of the EMG recordings, the contextual information was manually annotated.

### 2.2. Data Analysis and Preparation

#### 2.2.1. Class Labels

The classification models presented in this paper are intended to predict the grasp type. As shown in Table 2, through the variable GRASP_DH, the taxonomy of grasps in the database is based on nine types, one of them uncategorized (9 = Free) and, therefore, not used. Some grasps had very few recordings, while the cylindrical grasp represented 42% of the recordings. Considering that this grasp is also the most commonly used by humans in ADL [28], the classification problem was reduced to a binary task aimed at detecting cylindrical grasping: cylindrical class versus non-cylindrical class (all other classes).

#### 2.2.2. EMG Data

From the original dataset with around 15,800 EMG continuous recordings, only ETs labeled as reaching have been used, as they are the most useful event for predicting hand grasp type for the application to advanced prosthetics, robotic hands or human–machine interaction systems. Luckily, the reaching events are the most frequent in the database, resulting in a total of 4573. The samples labeled as a “free” grasp type (which means that the users could grasp however they wanted) were removed. In addition, after visually inspecting some of the signals, it was observed that some samples contained null values in the beginning with few of them being completely null, most likely due to a lag in the recording process of the EMG (wireless) and the synchronization with kinematic records (wired). Thus, the null data were discarded and, as a result, only 4209 recordings were left to be used.

**Figure 2:**
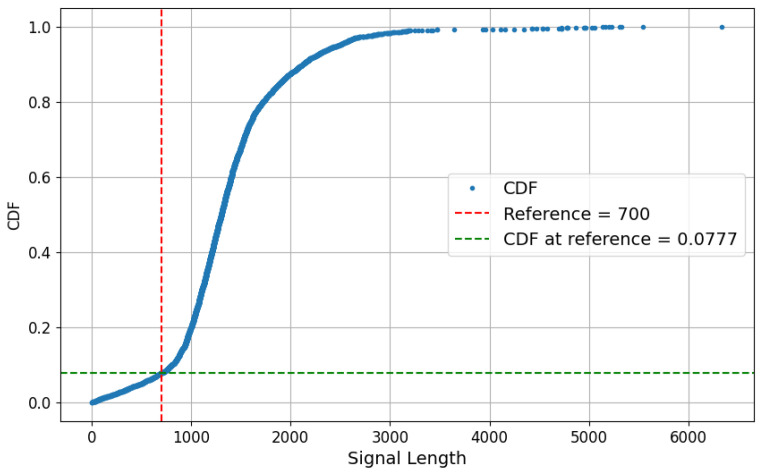
CDF with the proposed cut-off threshold (700 samples), along with the percentage of rejected samples (7.77%).

Afterwards, in order to eliminate clean signals that were too short to have sufficient discriminative information, a cumulative distribution function (CDF) was used to analyze the signal length distribution and determine a cut-off threshold. Figure 2 shows the CDF of trimmed signal lengths, indicating that a 700-frame threshold would retain 92.23% of signals with complete EMG features, while only 7.77% would be rejected. After applying this filter rule, the distribution of the final recordings remains at 42% for the cylindrical class and 58% for the rest. Thus, ultimately, 3883 recordings were used in this work.

Finally, due to the different task heights, object placements, etc. [24], the data length of the reaching phase for each recording is varied. Considering that some recordings were already trimmed and that the end of the signals most likely captures the grasping action, while the beginning may not reflect the hand movement accurately, the EMG data used in these experiments were adjusted to equal all signal lengths to ensure consistent training data length across all models. For that, the 3883 selected recordings were adjusted to a uniform length of 1000 frames. For recordings over 1000 frames, only the last 1000 frames were kept, as this final segment best represents the grasp type performed. For recordings under 1000 frames (but above 700), zeros were added at the beginning until they reached 1000 frames.

#### 2.2.3. Context Data

According to Fitts’ Law [29], the time required to complete a movement is influenced by the distance to the target and the size of the target, reflecting the trade-off between speed and accuracy in motor tasks. Based on this principle, the contextual variables that have been used (see in Table 2) are weight, task height, primary span (cross-section of the grasped zone) and secondary span (secondary grasping section) because they are expected to directly impact the registered tasks.

**Figure 3:**
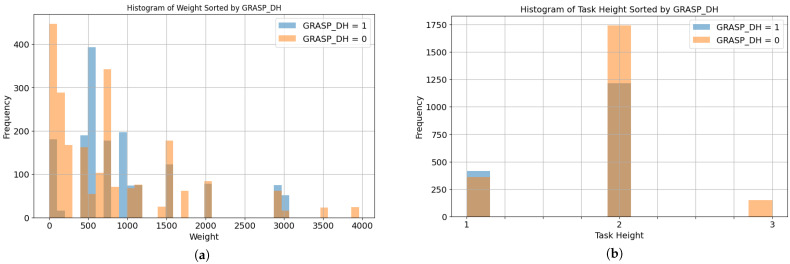
Data distributions by class and contextual data: (**a**) Weight. (**b**) Task height.

Figure 3a shows the distribution of the product weight for the two classes. As the majority of the cases are represented under 1 kg of weight for both classes, the models are not expected to be biased to predict one class over the other based on the weight information alone. The task height distribution is depicted in Figure 3b, where for class 0, there is an over-representation of task height 2 and task height 3.

**Figure 4:**
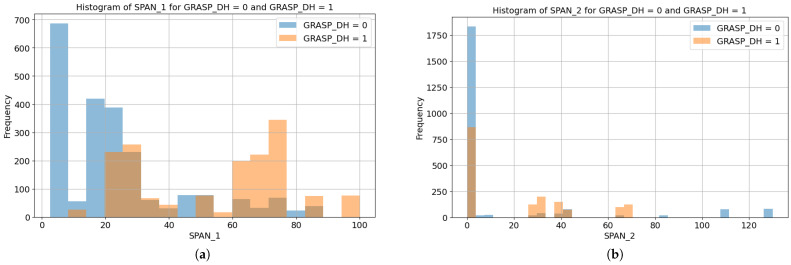
Comparison of SPAN distributions by class: (**a**) Span 1, main span of the product. (**b**) Span 2, secondary span of the product.

Finally, for the size of the products (Figure 4a), the SPAN_1 is distributed along a similar range of values for both classes. In the right plot, it can be appreciated that for both labels, most cases do not have information on the SPAN_2, which means that the product was only meant to be grasped by one cross-section (SPAN_1) which was labeled in the dataset.

The numerical variables in the contextual data (span and weight) were scaled between 0 and 1 using Min-Max normalization to preserve relationships between data points. The task height was one-hot encoded, as it is a categorical variable, so this variable was subdivided into three binary variables.

#### 2.2.4. Data Partitions

The data were split by participants to help the model generalize better, and to prevent it from learning the patterns of subjects that could introduce biases in grasp classification. For the train–test split, the 10 subjects with the lowest number of recordings were used for testing. Of the remaining 20 participants, the 4 participants with fewer recordings were chosen for validation, and the remaining 16 participants were used for training (see Table 3 for details).

### 2.3. Classification Models

The software used to develop this work includes MatLab R2023b, Python 3.11.9, Fastparquet 2024.0, PyArrow 17.0.0 and Keras 3.5 on TensorFlow 2.17.0.

#### 2.3.1. Unimodal Models

**Figure 5:**
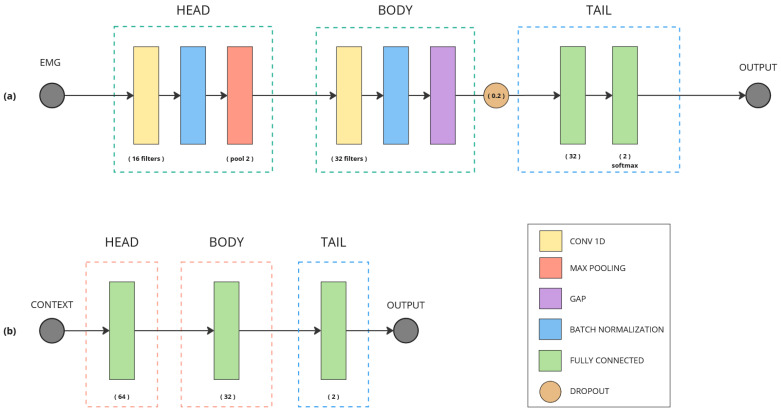
(**a**) CNN for EMG signals; (**b**) FC neural network for contextual data.

The single-input models are intended to set a baseline based on results with only one source of data as input in order to more objectively evaluate the performance of the multimodal approach. With this in mind, two simple architectures have been developed. Figure 5a shows the architecture for the EMG model (M_EMG). It is based on convolutional layers to process the temporal information of EMG signals, an approach that has been successfully used in the literature [30]. For the contextual model (M_CONTEXT) (Figure 5b), a very simple structure based on fully connected (FC) layers was designed. The complexity and final configuration of both models were determined by a manual hyperparameter exploration guided by the validation set.

#### 2.3.2. Multimodal Model

**Figure 6:**
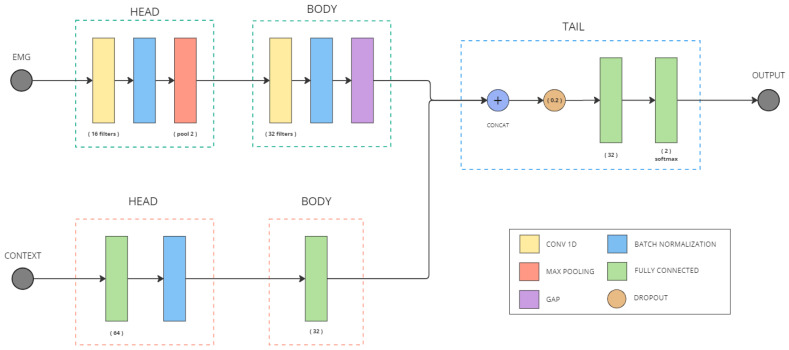
Hybrid model architecture (M_HYBRID).

The hybrid model (M_HYBRID) (Figure 6) combines the two previous models in two parallel branches. Then, the embeddings learned by both branches are concatenated. This extended feature space is reduced to a lower-dimensional representation space to extract the most important information from the concatenation, which is finally connected to the model output layer that generates the grasp prediction.

#### 2.3.3. Study of the Relevance of Contextual Variables

In order to evaluate the relevance of each context variable to the performance of the hybrid model, systematic experiments were performed by excluding variables one by one to the base M_HYBRID model.

#### 2.3.4. Hyperparameter Setup

After exploring the hyperparameter space using the validation set as a selection criterion, the model setup for all of the experiments was as follows:

- Optimizer: ADAM
- Loss function: binary cross-entropy
- Activation function of hidden layers: ReLU
- Activation function of output layers: softmax
- First convolutional layer: number of filters = 16, kernel size = 5
  (these filters operate over the 7 EMG channels)
- Second convolutional layer: number of filters = 32, kernel size = 5
- Learning rate scheduler: cosine decay (alpha = 0.01 initial learning rate = 0.001)
- Epochs: 300
- Batch size: 68

#### 2.3.5. Classification Metrics

Considering the cylindrical class as the positive class, the classification metrics used in this work are the proportion of correct predictions over all the samples (accuracy), the ratio of positive predictions over predicted positives (precision), and the true positive rate (recall). These metrics are derived from the confusion matrix, which summarizes the performance of a classification model by tabulating true positives, true negatives, false positives, and false negatives. These metrics are appropriate for describing and detecting possible biased behavior of classification models.

## 3. Results

After the signal preprocessing was completed as explained in the previous section, the classification models were trained and evaluated based on the methodology described in Section 2.3. The results are structured as follows: Section 3.1 compares unimodal and multimodal models, presenting training and validation results (Section 3.1.1) describes the test performance (Section 3.1.2), and provides an overall comparison (Section 3.1.3). Section 3.2 examines the relevance of contextual variables, according to the approach described in Section 2.3.3.

### 3.1. Unimodal Versus Multimodal Models

#### 3.1.1. Training and Validation Results

**Figure 7:**
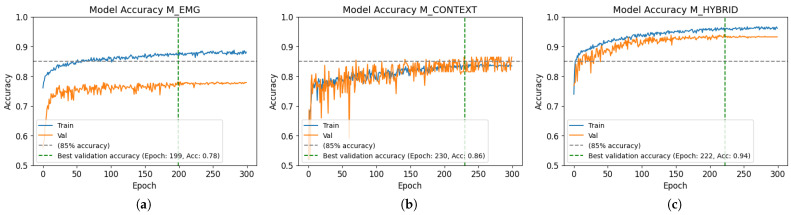
Training results of the models: (**a**) EMG, (**b**) contextual, (**c**) hybrid.

The training results in terms of the accuracy of the three main models are disclosed in Figure 7. The learning curves for the training and validation sets show that the model that reached the highest validation accuracy was the multimodal model. Although the model based on EMG reached the best validation accuracy in the shortest amount of time, the gap between both curves (train and validation) indicates overfitting. The plots for the contextual and hybrid models show less overfitting and the highest validation accuracy was obtained at epochs 230 and 222, respectively. The reference line set at 85% accuracy points out that the EMG model never reaches this level of the metric for the validation set, while the other two do. Furthermore, the hybrid model converges much faster and arrives at this value in the first epochs, and clearly shows the best performance of the three models.

#### 3.1.2. Test Results

**Figure 8:**
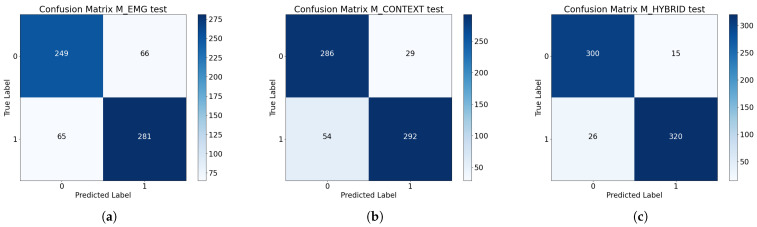
Confusion matrices of the models: (**a**) EMG, (**b**) contextual, (**c**) hybrid.

In the test phase, the unimodal models achieve 80% accuracy and 0.49 loss for the EMG and 87% for the contextual data with a loss of 0.34. The multimodal approach reaches 94% accuracy and 0.19 loss. The hybrid model was significantly more accurate than both unimodal models, outperforming the EMG- and context-based models by approximately 14 and 7 percentage points, respectively. In the confusion matrices (Figure 8b), the contextual model has a bias towards predicting the negative class 0 (non-cylindrical grasp), which can be seen from the high number of false negatives (54). However, the M_HYBRID model (Figure 8c) shows a more balanced misclassification rate. Similarly, the EMG model has more misclassifications, but they are evenly distributed.

#### 3.1.3. Comparison Overview

Table 4 summarizes the full display of the above metrics for the training, validation and test sets for the three models. The relationships between the three metrics for the three models show that although M_EMG reaches consistent accuracy and precision values during training, the recall is quite low, so it is missing some true-positive cases. In validation and test results there is a drop in accuracy and precision, so all the metrics are balanced and the misclassifications are balanced, too. For the M_CONTEXT model, the slightly higher values for precision against recall explain the bias towards predicting the majority negative (non-cylindrical). In contrast, the hybrid model demonstrates improved and more balanced performance across all metrics, suggesting that the integration of both modalities enhances the overall robustness of the classification.

### 3.2. Study of the Relevance of Contextual Variables

**Figure 9:**
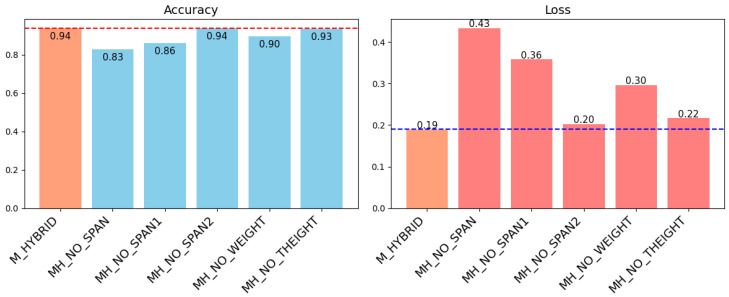
Comparison of accuracy and loss for hybrid models. The dashed lines indicate the best values achieved.

Based on an analysis that involved excluding each of the variables of the multimodal model, Figure 9 shows the classification results on the test set. Table 5 summarizes the results on the test set for the three metrics considered. The model M_EMG and the models without information of the SPAN1 showed the highest loss. The model that outperformed all others was the hybrid including all of the contextual variables, although the models MH_NO_SPAN2 and MH_NO_THEIGHT maintained similar levels of all metrics.

## 4. Discussion

The models analyzed in this study were developed based on a carefully curated dataset, following standard recommendations to ensure data quality and reliability. This curation process was essential in constructing robust models capable of capturing meaningful patterns. Furthermore, the dataset used represents real ADL rather than being limited to posture-based information. This approach allows the models to learn from real-world activity dynamics.

### 4.1. Unimodal Versus Multimodal Models

Among the unimodal models (Figure 5), the contextual model accuracy outperforms the EMG model by 7% but, when the two data sources are combined, the hybrid model is able to outperform both of them by 7% and 14%, respectively. Not only that, but when comparing the model losses, the hybrid model reduces the loss by more than 44% compared to the context-based model, which ensures that multiple data modes help improve generalization, increasing prediction confidence.

As seen in Figure 7, both unimodal models showed significant learning deficiencies. In terms of overfitting, the worst model was the EMG-based model, which did not generalize well on validation and test data, even with the MVC normalization of records that was expected to counteract the subjects’ variations. The EMG data used in this work represent real reaching actions with different objects variable in size and weight, followed by other actions. The literature suggests that the hand would be prefixed given the context and the future grasp, which should be represented in the EMG signals; however, they do not seem to be informative enough. This limitation likely stems from the inherent complexity of EMG signals, which are influenced by the variability of individual physiology, their non-linear nature, and their susceptibility to noise and artifacts. On the other hand, the unimodal contextual model showed a clear bias towards predicting the negative non-cylindrical class, as shown in Figure 8. Given the results of the multimodal model, the integration of both data modes leads to a solution that compensates for the flaws of unimodal models. That is, the context-aware EMG-based model showed a generalization ability far superior to those of the unimodal solutions, and a remarkably unbiased performance when making predictions on the unseen data of the test set.

In terms of performance (see in Table 4), the contextual model achieved an accuracy of 84% on the training set and 87% on the test set, while the reference threshold was set at 85% for comparison. Although the contextual model was able to reduce overfitting compared to the EMG model, its performance on the validation set remained below the 85% reference threshold.

As demonstrated in this work, context-aware models are designed to adapt to task-specific contextual conditions during processing, enhancing their ability to make accurate predictions. By integrating context as a first-class input, these models can refine predictions and decisions, yielding better outcomes than context-agnostic approaches.

### 4.2. Study of the Relevance of Contextual Variables

The performance of the different versions of the hybrid model based on excluding each contextual variable explains the relevance of the different data sources for the detection of cylindrical grasp. As highlighted in Figure 9, the best model in terms of loss on test data is the one that includes all of the characteristics. However, there is little difference between these results and the ones of the models in which task height and secondary span were removed. In the case of the task height, this characteristic could be discarded without a significant impact on model performance, probably because the majority of the tasks were developed in task height 2. A more representative set of this category could be retrieved to test this further.

As explained in Section 2.2.3 concerning Figure 4, SPAN_2 was registered as 0 for many of the recordings, as it was not used in many of the tasks; thus, it is expected to have less influence. However, the inclusion of the height of the product (longitudinal dimension, as both spans used in this work are cross-sectional) as a secondary span may also be relevant information that has not been explored. The secondary span has also proved to have some synergistic effect, since when both spans are removed, the performance of the model is worse than if only the main span is discarded (MH_NO_SPAN against MH_NO_SPAN1), with a difference in accuracy of 3%.

The main span was the variable with the greatest influence, as the model without it loses 8% of accuracy over the original model and it has the second highest loss (Figure 9). This variable was more evenly distributed for both classes, although class 0 tended to have smaller objects (in this category, there are grasps such as pinching motions that are used for products with a smaller span). The posture of the wrist can also differ depending on the selected grasp, thus affecting the EMG signal. There is another possible cause that can explain the influence of the grip width (SPAN_1) on the prediction success: the hand tries to preset itself with more or less opening, and this affects the flexor and extensor muscles of the fingers located in the forearm, which were used as a source of EMG.

The other variable with the greatest influence is the weight, as the loss increases by more than 57% and the accuracy of the model decreases by 4% (see Table 5). The weight, even the object is grasped, helps with prediction, as muscles will also activate with greater or lesser force depending on the physical characteristics of the object.

The exclusion of specific variables in the hybrid model led to a reduction in the total number of parameters, although the changes are relatively marginal. In particular, removing a single variable (as in MH_NO_SPAN1 or MH_NO_SPAN2) reduces the total number of parameters by approximately 0.8% (192 params, comparing M_HYBRID with MH_NO_SPAN1 and MH_NO_SPAN2). On the other hand, eliminating two variables simultaneously (MH_NO_SPAN) results in a 1.6% reduction (384 params, comparing M_HYBRID with M_HYBRID_N_SPAN). Finally, the exclusion of a different variable (MH_NO_THEIGHT, with three categories) generates a reduction of 2.4% (576 params).

While these reductions are marginal in our case, they illustrate a desirable effect in terms of model simplification. In future studies with more advanced architectures and more complex datasets, this impact could be more significant, requiring further analysis of the influence of variable selection on model efficiency.

## 5. Conclusions and Future Directions

Grasping is a fundamental aspect of daily life, and improving predictive models for grasp types is essential for advancing prosthetics, robotics, and human–machine interaction. This study explored multimodal grasp prediction by integrating EMG signals with contextual information and tested the importance of each contextual source.

Overall, it could be said that the addition of contextual data created a synergy with the EMG source that increased the chances of prediction. The most influential variables were the size of the cross-section of the object and the weight, although all pf the variables contributed towards the results.

Although the simplicity of the proposed model has proven to be effective, it needs to be expanded to include additional grasp types, moving beyond the binary task. The possibility of implementing innovations such as hierarchical embeddings or attention mechanisms is being studied. The use of these sophisticated techniques that focus on the important parts of the data during the analysis could help our model with the overfitting that has been detected through the EMG source, since even with regularization techniques, it was not possible to avoid this effect.

However, the benefits of this simple network could be used to control a prosthesis using the other arm or the residual muscle (if amputation level is quite distal) as a source of EMG, or a HE using the same setup. As the sensors are placed in the forearm, the extraction of data in real-life scenarios is less invasive for the subjects than other proposals. In future studies, we aim to validate this model with prostheses or other devices using additional EMG recordings for robustness.

Following the previous research, an innovation that could be explored is the number and placement of the EMG sensors. The extension of the area to the upper arm or the shoulder will be considered to analyze the performance of the models with different placements with richer physiological data. The number of sensors can also be reduced to the proximal half of the forearm to study the need for a high number of electrodes. As already mentioned, although the forearm placement causes less discomfort to the user, the minimum number of EMG sensors will increase the acceptance of these types of devices.

Some more advanced modules could be implemented to extract the contextual data, such as a vision module capable of interpreting the scene. The automatization of this data extraction process could be achieved with a camera and a real-time vision module to detect the objects through well-established solutions such as the YOLO models [31]. After object detection, the contextual features could be extracted by estimating object properties from both RGB images and depth maps. In this case, it is expected that the longitudinal dimension of the object could serve as an estimation of the weight, which has been proven to work well.

Additionally, a CLIP-based [32] module could enhance contextual understanding by associating detected objects with semantic information derived from image-text embeddings. This approach would enable a richer interpretation of the scene, allowing the model to infer object affordances or likely usage scenarios based on textual descriptions and prior knowledge. By combining YOLO for precise object localization with CLIP for semantic reasoning, the system could improve grasp type predictions by leveraging both visual and contextual cues more effectively.

The integration of cameras in prosthetic systems has already been explored in various studies [33,34]. Inspired by these approaches, a vision-based module could improve grasp-type recognition. While high-performance models like YOLO offer precise object detection, these approaches could also guide the development of a lightweight, task-specific vision model optimized for prosthetic applications [35].

Beyond vision-based methods, other contextual information could be incorporated through alternative modalities. For instance, the user could verbally describe the task and the objects involved, allowing a speech recognition module to interpret this information and refine grasp selection accordingly.

Another opportunity to enhance the model could be the integration of Fitts’ Law to predict the difficulty of grasping based on the object’s size and distance, helping to guide grasp type selection in a straightforward manner for prosthetic devices. In addition, incorporating the kinematic data that already exist in the dataset, which provide real-time feedback on the hand’s position, velocity, and movement efficiency, alongside EMG signals, could further refine this process. By considering both physiological (EMG) and behavioral (kinematic) data, we could build a multi-modal, more responsive system that better mimics human grasping behavior, making it more user-friendly and adaptive to a variety of tasks. This approach could be used in devices where kinematic data are available, such as HEs.

Given the positive results of the analyses in this work, we aim to develop an advanced model capable of accurately predicting several grasp types beyond the binary dichotomy and introduce innovative modelling techniques that help boost its performance.

## Figures and Tables

**Table 1:** Task breakdown with elementary tasks and actions.

Task	Elementary Task	Action
Transporting a plastic bottle of water	Move closer to take the object from the top shelf	Reaching
	Take the object from the top shelf. Leave it on the kitchen top	Transportation
	Release the object and return to P1	Release
	Move closer to take the object from the kitchen top	Reaching
	Take the object from the kitchen top. Leave it on the bottom shelf	Transportation
	Release the object and return to P1	Release
Plugging a flat electric plug	Push to put the plug in	Pushing
	Pull to take the plug out	Pulling
	Hold for 2 seconds	Holding

**Table 2:** Description of the database variables used in this paper.

Variable	Description	Original Range		New Range
ID	Unique identifier for each recording used for training the models.	–		–
PARTICIPANT	Numeric identifier for the participant performing the task.	Values: 1–30		–
T (Task)	Combination of movements that each participant performed. One participant can have the same task several times, as there are several elementary tasks for each task.	Values: 102–180, 202–234, 302–350		–
ET (Elementary Task)	Each movement that the participant performed in each task.	Values: 1–614		–
datasize	Number of EMG frames for each recording.	41–6337		1000 ^1^
PRODUCT_DH	Identifier of the product grasped with the dominant hand.	Values: 1–105		–
WEIGHT ^2^	Weight of the product.	In grams		[0, 1] ^1^
SPAN_1/SPAN_2 ^2^	Width of the part of the object where it is grasped. If the object has two possible parts (e.g., a water bottle), both spans are specified, in other case SPAN_2 = 0.	In mm		[0, 1] ^1^
GRASP_DH	**LABEL TO PREDICT. Grasp type performed with dominant hand.**	1 = Cylindrical 2 = Lateral pinch 3 = Lumbrical 4 = Oblique 5 = Special Pinch	6 = Hook 7 = Intermediate 8 = Five finger pinch 9 = Free	0 = Non-cylindrical (classes 2–8) 1 = Cylindrical ^1^
ACTION_DH	Action performed with dominant hand.	1 = Reaching 2 = Releasing 3 = Transporting 4 = Holding 5 = Pouring	6 = Pulling 7 = Pushing 8 = Twisting (clockwise) 9 = Twisting (anticlockwise) 10 = Other	Only using 1 (reaching)
TASK_HEIGHT ^2^	Height of task performance.	1 = High–Median, 2 = Median, 3 = Median–Low		One-hot encoded

^1^ See Section 2.2 for details. ^2^ These variables have been directly used as input for contextual and hybrid models.

**Table 3:** Data distribution for classification experiments.

Group	Number of Subjects	Recordings (% of Data)
Training	16 (53.3%)	2720 (70%)
Validation	4 (13.3%)	502 (13%)
Testing	10 (33.3%)	661 (17%)

**Table 4:** Metrics of unimodal and multimodal approaches for training (tra), validation (val) and test.

Metrics	M_EMG			M_CONTEXT			M_HYBRID		
	Tr	Val	Test	Tr	Val	Test	Tr	Val	Test
Accuracy	0.90	0.78	0.80	0.84	0.86	0.87	0.98	0.93	0.94
Precision	0.92	0.75	0.81	0.78	0.86	0.91	0.98	0.92	0.96
Recall	0.81	0.79	0.81	0.83	0.85	0.84	0.97	0.93	0.92

**Table 5:** Assessment of the relevance of each context variable on the performance of the hybrid model.

Model	Loss	Accuracy	Precision	Recall
M_EMG	0.49	0.80	0.81	0.81
M_CONTEXT	0.34	0.87	0.91	0.84
M_HYBRID	**0.19**	**0.94**	**0.96**	**0.92**
MH_NO_SPAN	0.43	0.83	0.84	0.84
MH_NO_SPAN1	0.36	0.86	0.89	0.83
MH_NO_SPAN2	**0.20**	**0.94**	0.94	**0.94**
MH_NO_WEIGHT	0.30	0.90	0.92	0.88
MH_NO_THEIGHT	0.22	0.93	**0.96**	**0.92**

## Data Availability

The original data presented in the study are openly available in [36] and the source code used to process the data and the models can be found at https://doi.org/10.5281/zenodo.14608801.

## Abbreviations

The following abbreviations are used in this manuscript:

EMG	Electromyography
HEs	Hand exoskeletons
CNN	Convolutional Neural Network
FE	Feature Extraction
ADL	Activities of Daily Life
MVC	Maximum Voluntary Contraction
T	Task
ET	Elementary Task
CDF	Cummulative Distribution Function
FC	Fully Connected
M_EMG	Model based on EMG data input
M_CONTEXT	Model based on contextual data input
M_HYBRID	Multimodal model
MH_NO_SPAN	Multimodal model without span variables
MH_NO_SPAN1	Multimodal model without span 1 variable
MH_NO_SPAN2	Multimodal model without span 2 variable
MH_NO_THEIGHT	Multimodal model without task height variable
MH_NO_WEIGHT	Multimodal model without weight variable

## References

[1] Corbetta D., Santello M. (2018). Reach-to-Grasp Behavior: Brain, Behavior, and Modelling Across the Life Span.

[2] Seegelke C., Hughes C.M., Knoblauch A., Schack T. (2013). Grasp posture planning during multi-segment object manipulation tasks — Interaction between cognitive and biomechanical factors. Acta Psychol..

[3] Feix T., Bullock I.M., Dollar A.M. (2014). Analysis of Human Grasping Behavior: Object Characteristics and Grasp Type. IEEE Trans. Haptics.

[4] Guo L., Lu Z., Yao L. (2021). Human-Machine Interaction Sensing Technology Based on Hand Gesture Recognition: A Review. IEEE Trans. Hum.-Mach. Syst..

[5] Pérez-Reynoso F., Farrera-Vazquez N., Capetillo C., Méndez-Lozano N., González-Gutiérrez C., López-Neri E. (2022). Pattern Recognition of EMG Signals by Machine Learning for the Control of a Manipulator Robot. Sensors.

[6] Li W., Shi P., Yu H. (2021). Gesture Recognition Using Surface Electromyography and Deep Learning for Prostheses Hand: State-of-the-Art, Challenges, and Future. Front. Neurosci..

[7] Xiong D., Zhang D., Zhao X., Zhao Y. (2021). Deep Learning for EMG-based Human-Machine Interaction: A Review. IEEE/CAA J. Autom. Sin..

[8] Noronha B., Accoto D. (2021). Exoskeletal Devices for Hand Assistance and Rehabilitation: A Comprehensive Analysis of State-of-the-Art Technologies. IEEE Trans. Med Robot. Bionics.

[9] Sultana A., Ahmed F., Alam M.S. (2023). A systematic review on surface electromyography-based classification system for identifying hand and finger movements. Healthc. Anal..

[10] Chowdhury R.H., Reaz M.B.I., Ali M.A.B.M., Bakar A.A.A., Chellappan K., Chang T.G. (2013). Surface Electromyography Signal Processing and Classification Techniques. Sensors.

[11] Hargrove L., Englehart K., Hudgins B. (2008). A training strategy to reduce classification degradation due to electrode displacements in pattern recognition based myoelectric control. Biomed. Signal Process. Control.

[12] Simar C., Colot M., Cebolla A.M., Petieau M., Cheron G., Bontempi G. (2024). Machine learning for hand pose classification from phasic and tonic EMG signals during bimanual activities in virtual reality. Front. Neurosci..

[13] Siu H.C., Shah J.A., Stirling L.A. (2016). Classification of Anticipatory Signals for Grasp and Release from Surface Electromyography. Sensors.

[14] Batzianoulis I., El-Khoury S., Pirondini E., Coscia M., Micera S., Billard A. (2017). EMG-based decoding of grasp gestures in reaching-to-grasping motions. Robot. Auton. Syst..

[15] Batzianoulis I., Krausz N.E., Simon A.M., Hargrove L., Billard A. (2018). Decoding the grasping intention from electromyography during reaching motions. J. NeuroEngineering Rehabil..

[16] Wang Z., Xiong C., Zhang Q. (2024). Enhancing the online estimation of finger kinematics from sEMG using LSTM with attention mechanisms. Biomed. Signal Process. Control.

[17] Gandolla M., Ferrante S., Ferrigno G., Baldassini D., Molteni F., Guanziroli E., Cottini M.C., Seneci C., Pedrocchi A. (2017). Artificial neural network EMG classifier for functional hand grasp movements prediction. J. Int. Med Res..

[18] Zandigohar M., Han M., Erdoğmuş D., Schirner G. (2020). Towards Creating a Deployable Grasp Type Probability Estimator for a Prosthetic Hand. Lecture Notes in Computer Science (Including Subseries Lecture Notes in Artificial Intelligence and Lecture Notes in Bioinformatics).

[19] Jaramillo-Yánez A., Benalcázar M.E., Mena-Maldonado E. (2020). Real-Time Hand Gesture Recognition Using Surface Electromyography and Machine Learning: A Systematic Literature Review. Sensors.

[20] Coskun M., Yildirim O., Demir Y., Acharya U.R. (2022). Efficient deep neural network model for classification of grasp types using sEMG signals. J. Ambient Intell. Humaniz. Comput..

[21] Atzori M., Gijsberts A., Castellini C., Caputo B., Hager A.G.M., Elsig S., Giatsidis G., Bassetto F., Müller H. (2014). Electromyography data for non-invasive naturally-controlled robotic hand prostheses. Sci. Data.

[22] Pizzolato S., Tagliapietra L., Cognolato M., Reggiani M., Müller H., Atzori M. (2017). Comparison of six electromyography acquisition setups on hand movement classification tasks. PLoS ONE.

[23] Mora M.C., García-Ortiz J.V., Cerdá-Boluda J. (2024). sEMG-Based Robust Recognition of Grasping Postures with a Machine Learning Approach for Low-Cost Hand Control. Sensors.

[24] Roda-Sales A., Jarque-Bou N.J., Bayarri-Porcar V., Gracia-Ibáñez V., Sancho-Bru J.L., Vergara M. (2023). MOVMUS-UJI Dataset & ERGOMOVMUS: EMG and kinematics data of the hand in activities of daily living with special interest for ergonomics. Zenodo.

[25] Jarque-Bou N.J., Vergara M., Sancho-Bru J.L., Roda-Sales A., Gracia-Ibáñez V. (2018). Identification of forearm skin zones with similar muscle activation patterns during activities of daily living. J. NeuroEng. Rehabil..

[26] Hermens H.J., Freriks B., Disselhorst-Klug C., Rau G. (2000). Development of recommendations for SEMG sensors and sensor placement procedures. J. Electromyogr. Kinesiol..

[27] Konrad P. (2005). The ABC of EMG: A Practical Introduction to Kinesiological Electromyography.

[28] Vergara M., Sancho-Bru J.L., Gracia-Ibáñez V., Pérez-González A. (2014). An introductory study of common grasps used by adults during performance of activities of daily living. J. Hand Ther..

[29] Fitts P.M. (1954). The information capacity of the human motor system in controlling the amplitude of movement. J. Exp. Psychol..

[30] Shen S., Gu K., Chen X.R., Yang M., Wang R.C. (2019). Movements Classification of Multi-Channel sEMG Based on CNN and Stacking Ensemble Learning. IEEE Access.

[31] Terven J., Córdova-Esparza D.M., Romero-González J.A. (2023). A Comprehensive Review of YOLO Architectures in Computer Vision: From YOLOv1 to YOLOv8 and YOLO-NAS. Mach. Learn. Knowl. Extr..

[32] Radford A., Kim J.W., Hallacy C., Ramesh A., Goh G., Agarwal S., Sastry G., Askell A., Mishkin P., Clark J. Learning Transferable Visual Models From Natural Language Supervision. Proceedings of the 38th International Conference on Machine Learning.

[33] He Y., Shima R., Fukuda O., Bu N., Yamaguchi N., Okumura H. (2019). Development of distributed control system for vision-based myoelectric prosthetic hand. IEEE Access.

[34] Shi C., Yang D., Zhao J., Liu H. (2020). Computer Vision-Based Grasp Pattern Recognition with Application to Myoelectric Control of Dexterous Hand Prosthesis. IEEE Trans. Neural Syst. Rehabil. Eng..

[35] Gong W. (2024). Lightweight Object Detection: A Study Based on YOLOv7 Integrated with ShuffleNetv2 and Vision Transformer. arXiv.

[36] Roda-Sales A., Jarque-Bou N.J., Bayarri-Porcar V., Gracia-Ibáñez V., Sancho-Bru J.L., Vergara M. (2023). Electromyography and kinematics data of the hand in activities of daily living with special interest for ergonomics. Sci. Data.

